# Engineering Islets From Stem Cells: The Optimal Solution for the Treatment of Diabetes?

**DOI:** 10.3389/fimmu.2022.869514

**Published:** 2022-04-27

**Authors:** Suya Du, Yanjiao Li, Zhen Geng, Qi Zhang, Leo H. Buhler, Carmen Gonelle-Gispert, Yi Wang

**Affiliations:** ^1^ Department of Clinical Pharmacy, Sichuan Cancer Hospital & Institute, Sichuan Cancer Center, School of Medicine, University of Electronic Science and Technology of China, Chengdu, China; ^2^ School of Medicine, University of Electronic Science and Technology of China, Chengdu, China; ^3^ Clinical Immunology Translational Medicine Key Laboratory of Sichuan Province, Center of Organ Transplantation, Sichuan Academy of Medical Science and Sichuan Provincial People’s Hospital, Chengdu, China; ^4^ Institute of Organ Transplantation, Sichuan Provincial People's Hospital, University of Electronic Science and Technology of China, Chengdu, China; ^5^ Chinese Academy of Sciences, Sichuan Translational Medicine Research Hospital, Chengdu, China; ^6^ Faculty of Science and Medicine, University of Fribourg, Fribourg, Switzerland

**Keywords:** stem cell, islet, diabetes, engineering, therapeutic efficacy

## Abstract

Diabetes is a metabolic disease characterized by insulin deficiency. Bioengineering of stem cells with the aim to restore insulin production and glucose regulation has the potential to cure diabetic patients. In this review, we focus on the recent developments for bioengineering of induced pluripotent stem cells (iPSCs), mesenchymal stem cells (MSCs), embryonic stem cells (ESCs), and pancreatic progenitor cells in view of generating insulin producing and glucose regulating cells for β-cell replacement therapies. Recent clinical trials using islet cells derived from stem cells have been initiated for the transplantation into diabetic patients, with crucial bottlenecks of tumorigenesis, post-transplant survival, genetic instability, and immunogenicity that should be further optimized. As a new approach given high expectations, bioengineered islets from stem cells occupies considerable potential for the future clinical application and addressing the treatment dilemma of diabetes.

## Introduction

Diabetes is one of the major public health challenges of the 21st century and bears heavily to global health costs. An estimated number of 463 million adults (1 in 11) around the world are living with diabetes, and this number is projected to reach 700 million by 2045 (1). Type 1, type 2 and gestational diabetes mellitus are three main categories of diabetes, and type 2 diabetes remarkably accounts for around 90% of diabetes cases worldwide (1). Although lifestyle modification and pharmacotherapy are both efficient to treat type 2 diabetes, marked variability in outcomes still widely exists resulting in irregular monitoring, sub-optimal use of effective medicines and inevitable disease progression due to decline of β cell function. Therefore, innovative therapies are required to implement for delaying β cell lost, regeneration of endogenous β cell mass or replenishment of β cells with engineering islets from stem cells.

**Figure 1:**
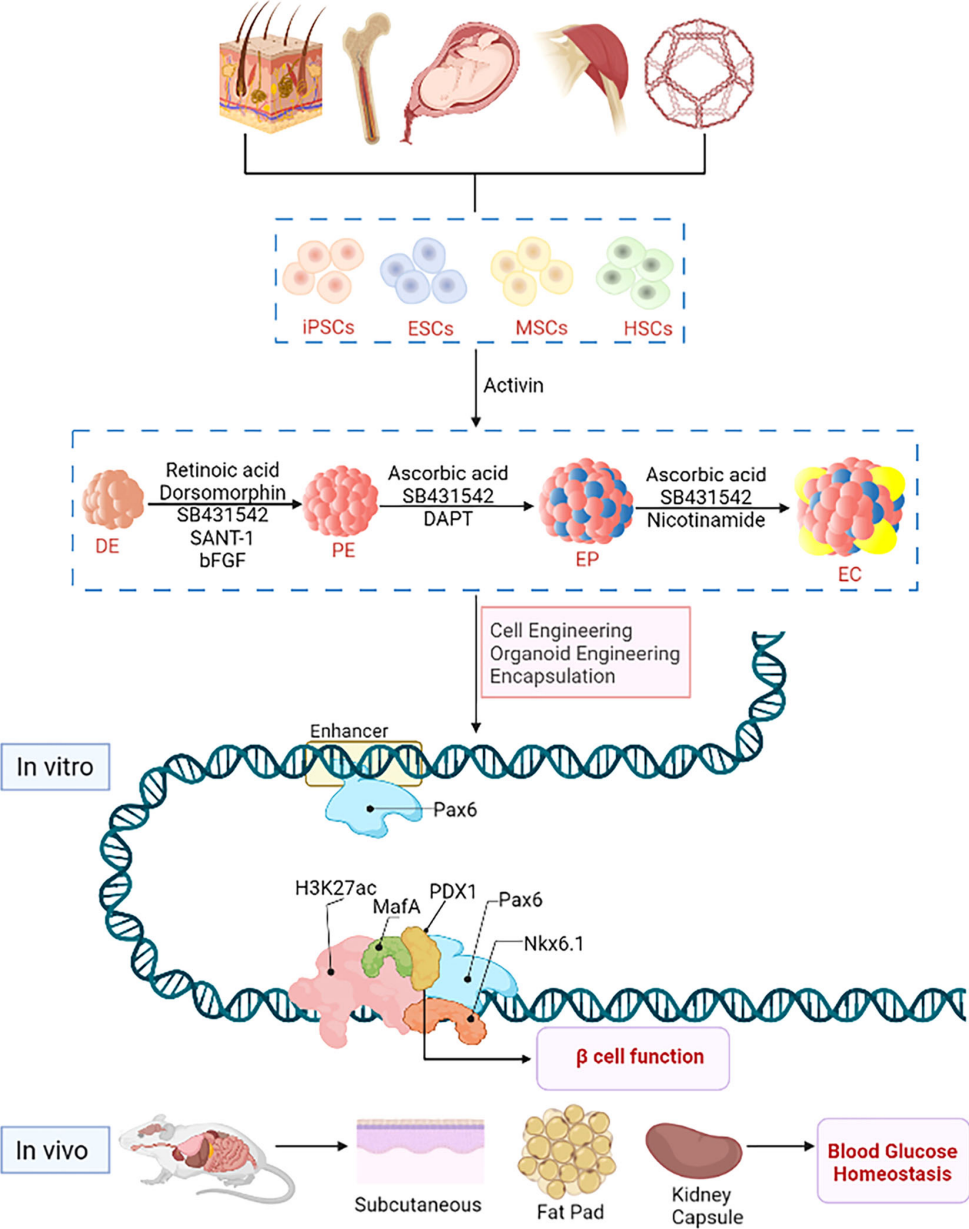
Stem cell engineering for type 1 diabetes. Stem cells originate from various sources, e.g. embryonic stem cells with strong ability of differentiation and self-renewal from embryonic tissues, hematopoietic stem cells and bone marrow stem cells from tissues or organs such as placenta, amniotic fluid, umbilical cord, bone, skeletal muscle, fat and skin; induced pluripotent stem cells are produced by genetic reprogramming of somatic cells. *In vitro*, predicted paired box 6 (PAX6) activates the expression of pancreatic β-cell-specific genes and proteins, such as pancreatic duodenal homeobox factor-1 (PDX-1) and NK6 Homeobox 1, (NKX6-1) and musculoaponeurotic fibrosarcoma oncogene family A (MafA), and interacts with these factors at the protein level to promote β-cell function. *In vivo*, hypoglycemic homeostasis is rapidly reestablished in diabetic immunodeficient mice (NOD/SCID) after transplantation with subcutaneous, renal capsule or fat pad.

Stem cells are undifferentiated cells with self-renewal and differentiation into various cell types (2–4). Since 1960s, scientists have successively identified and isolated hematopoietic stem cells (HSCs), bone marrow stem cells (MSCs), embryonic stem cells (ESCs) and developed induced pluripotent stem cells (iPSCs) (5). Thereinto, adult stem cells such as HSCs and MSCs are derived from bone marrow, skeletal muscle, fat, amniotic fluid, umbilical cord blood, skin, placenta and other tissues or organs, while embryonic stem cells are derived from embryonic tissues (6–9). IPSC are obtained through genetic reprogramming of somatic cells by ectopic expression of four transcription factors (OCt3/4, SOX2, C-MyC and Klf4) and have been generated from somatic cells such as mouse embryonic fibroblasts (MEF), adult mouse tail fibroblasts as well as human fibroblasts (10, 11) (**Figure 1**). Importantly, embryonic stem cells are totipotent and can differentiate into cell types derived from all three germ layers (12, 13). In contrast to embryonic stem cells, multipotent adult stem cells have limited self-renewal abilities and are prone to differentiate into specific adult tissue cells such as adipose tissue and muscle tissue (4).

With the continuous exploration of stem cells, an abundance of studies identified that cells such as MSCs and ESCs can grow indefinitely outside the body and maintain their ability to differentiate, which highlight their potentials as alternative sources of organ and tissue replacement (4, 14–16). Encouragingly, the results of emerging preclinical studies and clinical trials for diseases, such as diabetes have deepened our understanding of the use of stem cells in tissue engineering and cell therapy (17, 18). To date, new progress has been made in the treatment of brain diseases such as cerebral palsy (19–23), stroke (24–28), blood diseases (29), eye diseases (30–32) as well as diabetes (33–36), and the exploratory research on the treatment of diabetes with stem cells is developing in the right direction (36). In this review, we summarize the research field for stem cell differentiation and islet engineering, emphasizing on the efficacy of this new bioengineering technology applied for diabetes care, and elucidate current breakthroughs and future challenges of stem cell differentiation into islets.

## iPSCs-Derived Islet Cells for Islet Replacement Therapies

In recent years, it has been demonstrated that iPSCs have unlimited self-renewal ability and can be differentiated into multiple cell types such as neural stem cells (NSCs) (37), cardiomyocytes (38), dopaminergic neurons (39) and hepatocellular like cells (40). IPSC-derived islet cells might constitute a new source for islet cell replacement therapies (41). *In vitro*, most iPSC-derived cell lines initially express pancreatic and duodenal homeobox 1 (PDX1) and then further differentiate into PDX1, glucose transporter 2 (Glut2), musculoaponeurotic fibrosarcoma oncogene family A (MafA) and insulin expressing end-stage cells. One specific iPSCs cell line was detected to first express SOX17 and gradually express the β-cell-specific marker SOX9, PDX1 at later stages. The co-expression of C-peptide and PDX1 at a final stage confirmed the differentiation into insulin-producing cells (42). Additionally, it was shown that in order to obtain insulin-secreting cells *in vitro*, factors such as retinoic acid (RA), glutamine, noggin, nicotinamide and growth factors such as keratinocyte growth factor (KGF) and hepatocyte growth factor (HGF) are essential for the directed iPSCs differentiation (42, 43). Therefore, owing to the tremendous research potential of iPSCs in β cell replacement therapies, these stem cells are promising for further drug development and transplant medicine applications.

Furthermore, tissue engineering techniques such as 3D bio-printing have been substantially evolved since the 1980s for human therapeutic applications, including the creation of a bio-artificial pancreas. 3D bio-printing involves the isolation and expansion of human cells, followed by the automated printing of biodegradable scaffolds containing such cells. 3D bio-printed scaffolds are under investigation for various applications such as therapeutic devises, and *in vitro* model systems for analyzing diseases or screening drugs (44, 45). Undoubtedly, 3D bioprinting and regenerative medicine cooperatively hold great promise in building and assembling a bioartificial pancreas.

**Figure 2:**
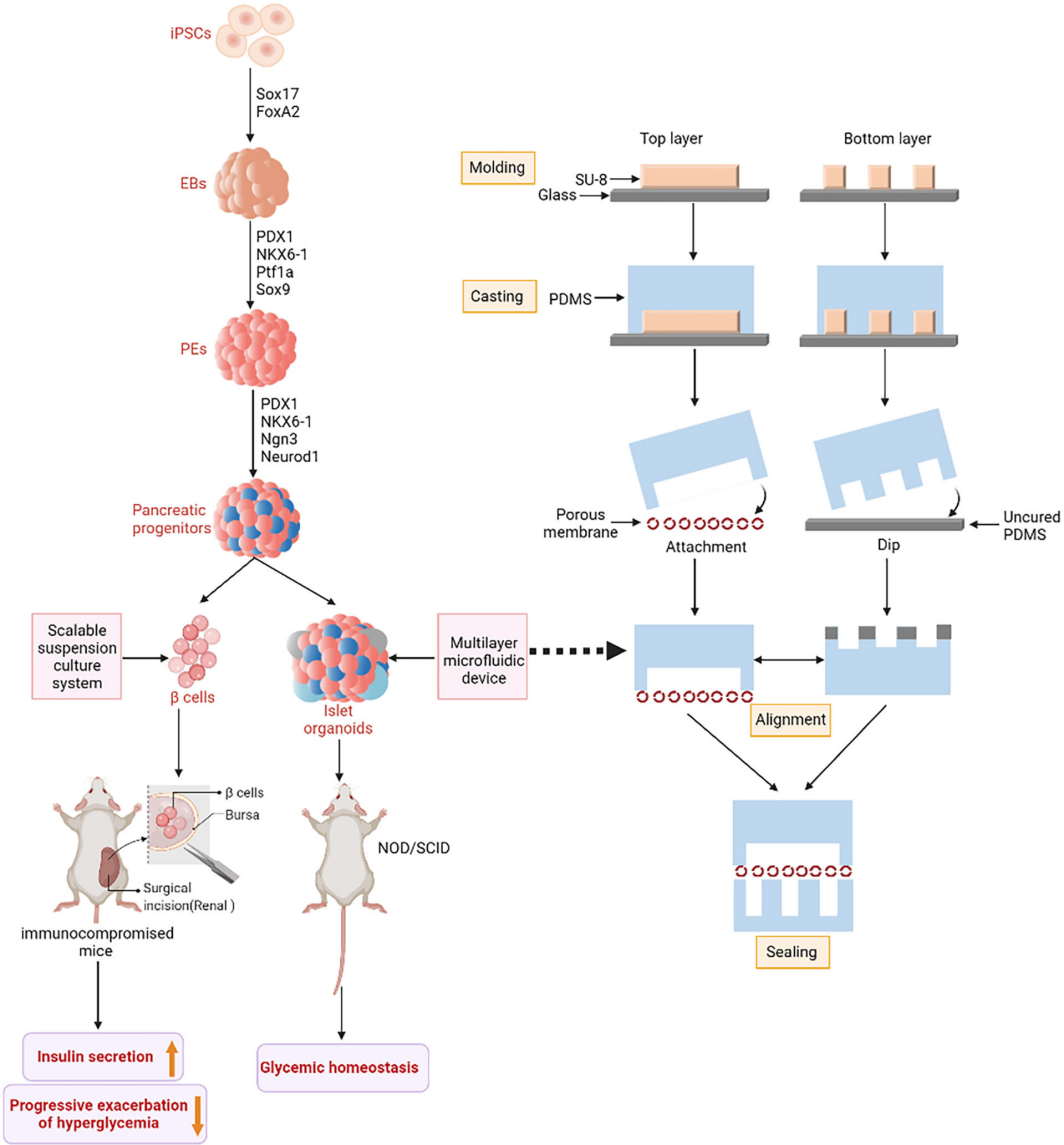
IPSCs-derived islet function replacement. iPSCs differentiate into insulin-secreting cells under the action of related transcription factors. Embryoid bodies (EBs) are formed under the influence of SLEy-related high mobility group box 17 (SOX17) and Forkhead box A2 (FoxA2). Pancreatic and duodenal homeobox 1 (PDX1), NK6 Homeobox 1 (NKX6-1), Pancreas Associated Transcription Factor 1a (PTF1a) and SOX9 guide to pancreatic Endoderms (PEs). Finally, PDX1, NKX6-1, Neurogenin 3 (Ngn3) and Neuronal Differentiation 1 (NeuroD1) differentiated into pancreatic progenitor cells. Pancreatic progenitor cells are directed to produce glucose-responsive β-cells. The figure on the right shows the fabrication method of microfluidic device. SU-8 photoresist is rotated onto two clean glass wafers and then selectively cured under ultra violet (UV) light using different masks. The mixture of Polydimethylsiloxane (PDMS) monomer and hardener then produces a two-layer copy of PDMS. Finally, the two PDMS replicas are sealed together with an intermediate porous membrane.

Organoids are defined as 3D multi-cellular spheroids obtained in *in vitro* cultures. Numerous 3D cell culture methods using islet cells had been described to obtain hetero cellular islet organoids (46). Remarkably, such hetero cellular islet organoids may integrate different types of supporting cells, such as endothelial cells, into insulin-producing structures, which is a valuable strategy to increase neovascularization of transplanted islets (46). Therefore, using human pluripotent stem cells to create organoids that resemble human pancreatic islets *in vivo* could help to overcome the organ scarcity. Herein, Tao et al. produced human islet organoids from human iPSCs using a perfusable organ on-chip system. The system integrated functional β-cells obtained after induction of endoderm, followed by differentiation and amplification of pancreatic progenitor cells and maturation of endocrine cells (**Figure 2**) (47, 48). As described in **Figure 2**, the islet-like organ was generated by step wise incubation with essential differentiation factors. Starting from primary embryoids (EBs), endodermal production was induced by activin, pancreatic final endodermal production was induced by dorsomorphin and RA, and finally the insulin producing pancreatic β-cell was induced by nicotinamide. This islet-like organ was generated under dynamic perfusion system, a multilayered microfluidic device composed of four parts top and bottom polydimethylsiloxane (PDMS) layers, through-hole PDMS membrane and polycarbonate porous membrane as separators (49). Fresh media was pumped through the upper and lower layers at 100µl per hour thereby providing continuous supply of media and nutrients for the formation and long-term culture of islet organs after EBs formation. In addition, produced islet-like organs contained heterogeneous islet-specific α- and β-like cells with sufficient cell viability. Simultaneously, during culture expression of pancreatic β-cell-specific genes and proteins such as PDX1 and NK homeobox 1 (NKX6-1), and C-peptide proteins related to insulin secretion were increased. Together, these results provide evidences that an islet-like organ generated through a perfusable islet-on-chip system is similar to the reproduction and development of human islets. And this technique offers a feasible and effective engineering method for generating functional islet-like organoids derived from iPSCs in a bionic microenvironment. Moreover, Yoshihara’ team also found that human iPSC-derived human islet-like organoids can rapidly restore glucose homeostasis after transplantation in diabetic immunodeficient mice (NOD/SCID) (50). The expression of immune checkpoint protein programmed death ligand 1 (PD-L1) in organoids was restored and glycemic homeostasis was maintained during 50 days in immunocompetent diabetic mice. Further, interferon-γ *ex vivo* exposure of human islets as well as human islet-like organoids derived from iPSC induced strong endogenous PD-L1 expression. Transplantation of PD-L1-overexpressing islet-like organoids into immunocompetent mice showed that iPSCs were protected from graft rejection in both allo- and xenotransplant settings. However, no studies on the transplantation of PD-L1-overexpressing islet-like organoids generated from iPSCs in the setting of the human immune system have been reported, which indicates the lack of evidence on determining PD-L1 expression profile for protecting human cells against the allogeneic human immune system. Meanwhile, this vacancy may greatly stimulate the progress of related research topics.

Another previous study described that glucose-responsive β-like cells can be efficiently produced by a scalable suspension culture system from ESCs and iPSCs *in vitro* (51). These stem cell-derived β-like cells (SC-β) expressed cytoplasmic C-peptide and nuclear protein NKX6-1, which is similar to islet β-cells. Other studies that transplanted human SC-β-cells into immune-compromised mice also showed β-cell functionality *in vivo*. Glucose challenges appeared after SC-β transplantation and human insulin in the blood was measured within weeks after transplantation in mice (17, 52, 53). Consequently, the cell transplantation under the renal capsule of immune deficient mice rapidly reversed the progressive exacerbation of hyperglycemia. Besides, 18 weeks after transplantation, it was remarkably observed that the mice receiving SC-β maintained normal human insulin secretion. Thus, SC-β transplantation successfully improved hyperglycemia in diabetic mice. As an advantage for clinical application, SC-β-cells can be produced from iPSCs of patients to avoid allogenic rejection after transplantation (51). In the future, autologous SC-β cell transplantation in combination with Treg adoptive immunotherapies may selectively suppress autoimmunity in patients with type 1 diabetes mellitus (T1DM), which could eliminate major obstacle for the cure of T1DM (54–56).

Despite the potential of iPSC to develop into a therapy for diabetic patients, important issues still need to be solved. The current limitations include: low reprogramming efficiency, reprogramming factors related to tumorigenesis, low survival and engraftment, loss of cell phenotype after transplantation, genetic instability, epigenomic instability and inherent immunogenicity need to be considered (57–61). Recently, a clinical case was reported describing the development of a teratoma in a diabetic patient after iPSCs-derived β cells transplantation (62). In this patient, β cells differentiated from autologous iPSCs were initially injected into the deltoid muscle where a mass with enlarged axillary lymph nodes were detected at two months after implantation. This tumor was characterized by rapid growth, local lymph node metastasis, more cellular atypia, and chemotherapy-resistance (62). As a milestone in regenerative medicine, iPSCs also shed new light on the treatment of age-related macular degeneration (AMD), one of the main causes of irreversible blindness. The researchers used the retinal pigment epithelium (RPE) cell sheets induced by autologous iPSCs from 2 patients with AMD through subretinal surgery. As a result, no serious adverse event was observed in 25 months of follow-up and no signs of rejection was noted without the administration of immunosuppressants in one patient, which provided valuable information on the feasibility and safety of iPSCs in the treatment of patients with macular degeneration and created a precedent for the clinical transformation of iPSCs in the field of regenerative medicine (63). *In vivo* tumorigenicity tests and a series of genomic analyses were both performed, although iPSCs-derived RPE cells had a low proliferation rate. In the iPSC-derived RPE cells obtained from Patient one, no genomic aberrations that were suggestive of tumorigenicity was found. However, three aberrations in DNA copy number (deletions) in iPSCs obtained from Patient two were detected, which could affect expression of genes encoded by both the deleted DNA and by DNA flanking the deletions (63). Based on the published information, the possible influence of these alterations on tumorigenicity could not be determined. In the reprogramming process to pluripotency and the cultivation of iPSCs, genetic instability was reported to be enhanced, potentially leading to additional genomic abnormalities (64). These genetic changes could negatively influence the performance and functional activities of iPSCs and increase tumorigenicity in replacing damaged tissues (65). Therefore, genomic stability must be maintained after reprogramming for further clinical uses.

As a renewable source of autologous cells, iPSCs have great prospects in regenerative medicine. It was generally accepted that autologous cells should be immune-tolerated by the recipient from whom the iPSCs are derived, whereas accumulating evidences remarkably revealed the rejections from autologous iPSC-derived cells, although the underlying related mechanisms remained controversial and still in the process of being gradually defined. As mentioned above, RPE cells derived from autologous iPSCs made the remission of AMD achievable. Even in non-ocular locations, they are also immune tolerated. However, smooth muscle cells (SMCs) produced from autologous iPSCs appeared to be significantly immunogenic, partly result from the abnormal expression of immunogenic antigens in iPSCs-derived SMCs (66). In C57BL/6 (B6) mouse transplantation model, immunogenic antigen-expressing B6 iPSCs and their differentiated target cells were immune tolerated under the kidney capsule but immune rejected when transplanted subcutaneously or intramuscularly owing to the lack of functional antigen presenting cells, indicating that the immune response toward antigens was also dependent on the immune environment of the transplantation site (67). Furthermore, autologous iPSCs and their derivatives were not inherently immunologically inert for autologous transplantation, due to *de novo* mutations in mitochondrial DNA (mtDNA) probably produced in the process of reprogramming to the iPSCs stage, long-term culture and differentiation into target cells. And these mtDNA mutations could encode neoantigens and elicit highly specific immunological response based on the host’s major histocompatibility complex genotype, which implied the indispensability to recheck the mtDNA mutations iPSC-derived products (68). Aberrant gene expressions in some cells generated from iPSCs can cause T cell-dependent immunological response in syngeneic recipients (69). Therefore, the immunogenicity of therapeutically valuable cells produced from patient-specific iPSCs should be assessed before clinic application in patients. In order to adopt the optimum immunosuppressive strategy to allow their engraftment, detailed evaluation of the inherent immunogenicity profiles of iPSC-derived somatic cell lineages is considerably required.

## ESCs-Derived β-Cells for Insulin Supplementation

**Figure 3:**
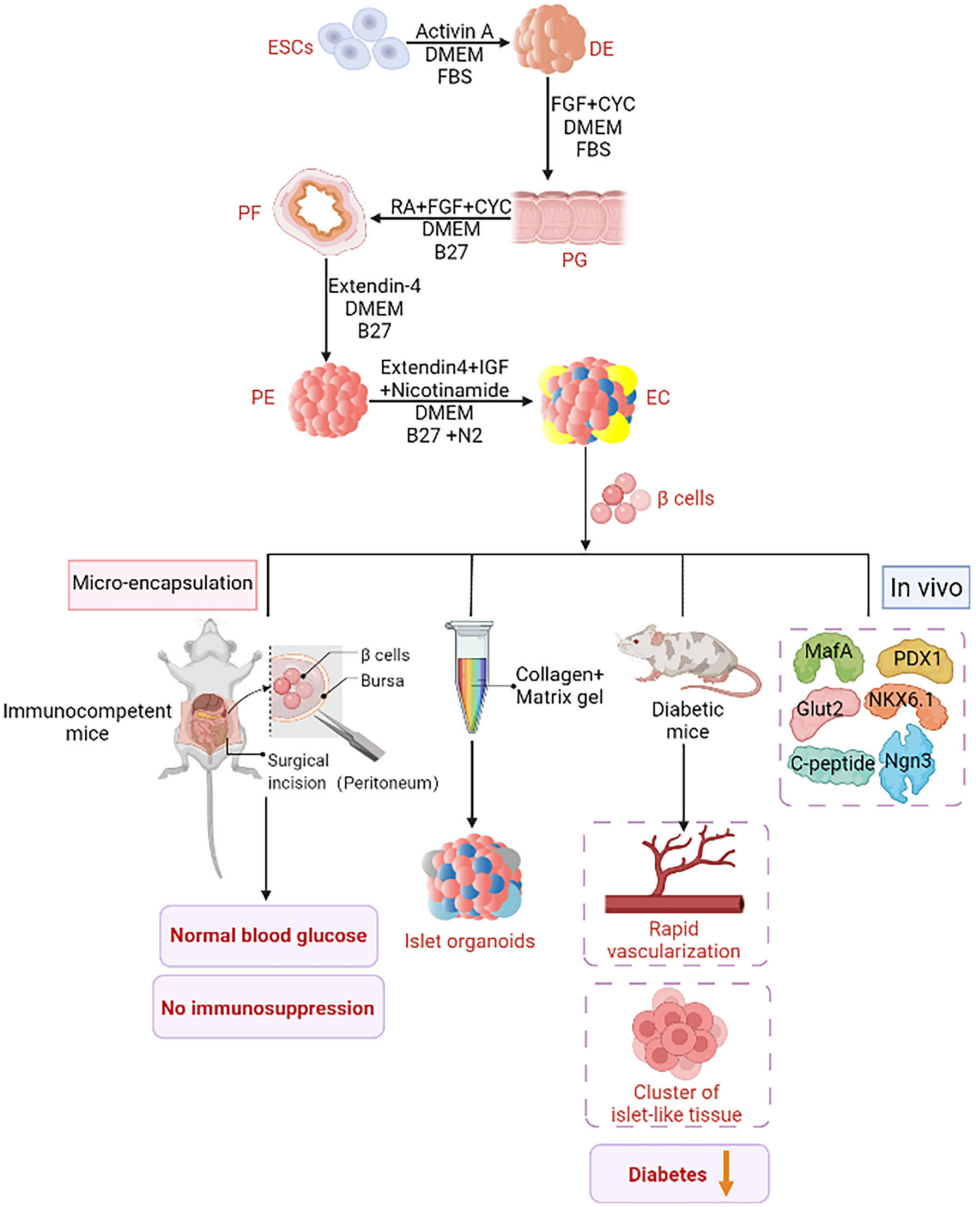
ESCs derived insulin supplement. An improved ESCs pancreatic differentiation protocol was developped. First, under the action of Activin A, DMEM, and FBS, one definitive endoderm (DE) was established through mesoderm. In the second step, activin A was removed and kaad-cycloamine (CYC) and fibroblast growth factor (FGF) were added to facilitate the transition from DE to the primitive gut tube (PG). The third step is to add retinoic acid (RA) and B27, and then to the posterior foregut endoderm (PF). Step 4: Extendin 4 (EX4) was added to support pancreatic lineage specific differentiation. The fifth step is to generate endocrine cells under the stimulation of EX4, IGF (insulin-like growth factor) and nicotinamide. These cells differentiate *in vitro* into insulin-secreting cells, Pancreatic and duodenal homeobox 1 (PDX1), neurogenin 3(Ngn3), MAF BZIP Transcription Factor A (MafA), glucose transporter 2 (Glut2), NK6 Homeobox 1 (NKX6-1) and C peptide were expressed. When these cells are injected into diabetic mice, diabetes is effectively ameliorated by rapid vascularization and the formation of a cluster of islet organoids. Islet organoids are obtained from ESCs by combining collagen and matrix gel in the biomimetic 3D scaffold. Islet sacking can quickly restore normal blood glucose without immunosuppression.

Insulin-secreting cells derived from pluripotent embryonic stem cells (ESCs) have emerged as one of the most attractive therapeutic alternatives for diabetes (70). Recently, it was shown that after transplantation of *in vitro*-differentiated stem cell-derived islets into immune-compromised mice, islets acquire a mature β-cell gene expression profile and can control blood glucose in the long term (71). Moreover, under adherent and suspension culture conditions, ESCs spontaneously differentiated into insulin-secreting cells *in vitro* with a very high proportion as observed through insulin immunohistochemical staining (72). A refined method for generating more mature insulin-producing cells from human ES has been described by Wang et al. using a three-dimensional differentiation culture. Most important steps for ES differentiation into insulin-secreting structures were first outlined by Lumelsky et al. (73) (**Figure 3**). These three-dimensional clusters were similar in structure to normal islets and contained all cell types of the endocrine pancreas (73). *In vitro* studies revealed that glucose as well as various secretagogues could trigger insulin release from these structures through similar mechanisms as in human islets. After transplantation into diabetic mice, these insulin-producing cells underwent rapid vascularization and formed clusters of islet-like tissue. Further, others showed that transplantation of islet cell clusters at stage 4 of differentiation, enriched of NKX6-1-expressing pancreatic progenitor cells through the action of a PKC activator, accelerates the maturation of insulin-secreting cells *in vivo*. Gradually, differentiation of human embryonic stem cells into islet cells has been widely used (74–76).

Solid evidence unveils that extracellular matrix (ECM) not only provides structural information for cells, but also plays a guiding role in cell development, which is crucial for maintaining tissue homeostasis and of great significance in embryogenesis, tissue-specific development and stem cell differentiation (77, 78). Cell-stromal interactions can promote β-cell proliferation (79, 80), insulin secretion (81, 82) and islet development (83, 84). Oberg-welsh and his group have demonstrated that ECM significantly enhances insulin secretion in fetal pig islet-like cell clusters *in vitro* (85). Besides, islet-like organs derived from human embryonic stem cells were successfully developed in a biomimetic 3D scaffold by combining collagen with matrix gel (86, 87). The resulting cell clusters included pancreatic α, β, and pancreatic polypeptide (PP) cells, but most of the resulting islet cells did not express glucagon, somatostatin, or PP. Expression of mature β-cell-marker genes such as PDX1, neurogenin 3 (Ngn3), insulin, MafA and Glut2 was detected in these 3D-induced cell clusters, whereas PDX1, NKX6-1 and C-peptide was highly expressed. Additionally, insulin secretory granules, indicating mature β-cells, were also detected. Although, neither collagen nor matrix gel materials are approved for clinical use, the results shed the light on the feasibility of generating islet-like organs from ESCs. Future breakthroughs by using supporting materials may lead to further progress.

A new transplantation strategy was proposed by Song and Millman who developed a large-pore recyclable 3D printing device composed of biocompatible poly-lactic acid (PLA) for subcutaneous transplantation of SC-β-cells (88). Clusters of SC-β-cells derived from human embryonic stem cells were embedded in biodegradable fibrin gel and inserted in the device. Severe transient hypoxia within the device that occurred after transplantation was mitigated by finite element modeling of cell oxygen concentration and evaluation of oxygen diffusion in different sized cell clusters embedded in hydrogel slabs. These adaptations allowed the device to be operated at physiological oxygen levels. After subcutaneous transplantation of the device into immune-compromised mice, SC-β-cells containing device was found to function for 12 weeks. Retrieved devices were structurally intact. Despite the observed host-tissue invasion, the mechanical strength and recyclability of such a device represent a considerable progress in the field. Other previous methods transplanting islets or cell clusters encapsulated in semipermeable microcapsules composed of alginate are challenged similarly by pericapsular fibrotic overgrowth (PFO) of microcapsules and the difficulty to retrieve grafts (89–91). Therefore, such a retrievable devise is promising for the application of SC-β-cells in regenerative medicine and serves as a platform for future transplantation strategies.

Insulin-producing cells derived from stem cells can address organ donor shortage, while cell encapsulation can reduce or eliminate the need for immunosuppression, minimizing the risks associated with islet transplantation procedures (92–97). Islet encapsulation provides a physical semi-permeable barrier not only preventing immune cell infiltration but also allows diffusion from necessary smaller molecules such as oxygen, nutrients, glucose, and insulin through the microcapsule. This is crucial for achieving widespread clinical use of the technique (98–100). Therefore, transplantation of microencapsulated stem cell-derived islets may extend islet transplantation to a larger cohort of patients. The embedding of immature β-cells derived from human embryonic stem cells into a sodium alginate hydrogel alleviated the response to foreign bodies *in vivo* and rapidly established normal blood glucose for 25 weeks after its transplantation into the peritoneum of immunocompetent mice (101). Vegas et al. reported long-term glycemic correction using human SC-β-cells in an animal model of diabetic immunocompetent mice (102). SC-β-cells coated with alginate derivatives and intraperitoneally implanted into streptozotocin (STZ)-treated C57BL/6J mice corrected blood glucose levels for 174 days during follow up. Retrieved implants still contained viable insulin-producing cells and showed minimal fibrotic overgrowth. In addition, a novel encapsulation approach was reported by the group of Alice A Tomei, in which transplantation of conformal coated islets, from fully MHC-mismatched Balb/c mice, achieved long-term (>100 days) survival after transplantation into epididymal fat pad or mammary fat pad of diabetic immune competent C57BL/6 mice (103, 104). Conformal coating minimizes capsule thickness, complies with islet shape, reduces transplant volume compared to encapsulated islets and allows glucose stimulated insulin release *in vitro* without delay (105). Moreover, when SC-β-cells were transplanted into gonadal fat pad of diabetic immunodeficient NOD-SCID mice, it was found that both uncoated and conformal coated SC-β-cells reversed diabetes. Blood glucose levels were maintained at normal levels for more than 80 days as obtained with human islets, demonstrating thereby safety and efficacy of this β-cell replacement strategy (103). To date, a small number of encapsulation systems have been used clinically with obvious safety (106). For example, βAir devices are designed for clinical use to ensure oxygen levels necessary for maximum islet function while microcapsules remain in the body. The device consists of two main components, an alginate saline gel plate containing islet modules and a gas chamber. After subcutaneous implantation, islets are oxygenated daily. Islet cells in the lumen absorb oxygen through diffusion *via* the permeable membrane (107). Remarkably, one case report describes a patient whose islets remained fully functional during a 10-month study period (108). Apart βAir devices, Theracyte and Sernova Cell Pouch also offers clinical devices which allows for pre-vascularization prior to implantation (106, 109).

In addition, Vertex Pharmaceuticals Incorporated recently announced unprecedented and positive Day 90 data for the first patient achieving successful engraftment and substantial improvement of islet cell function from VX-880, an novel investigational embryonic stem cell-derived and fully differentiated pancreatic islet cell replacement therapy for the treatment of type 1 diabetes (110). In this Phase 1/2 clinical trial, VX-880 was generally well tolerated with significant improvements in multiple measures, including fasting and peak stimulated C-peptide, HbA1c, and daily exogenous insulin requirement dose (111, 112). These unprecedented results introduce a potentially transformative medicine and deliver a life-changing therapy for T1DM patients and confer the remarkable promotion on the following VX-880 clinical studies, although there are still uncertain problems that need to be further clarified, including unexpected side effects, whether the treatment effectiveness would last a lifetime, and whether repeated treatment would be necessary.

## MSCs and Islet Co-transplantation for Islet Function Protection

**Figure 4:**
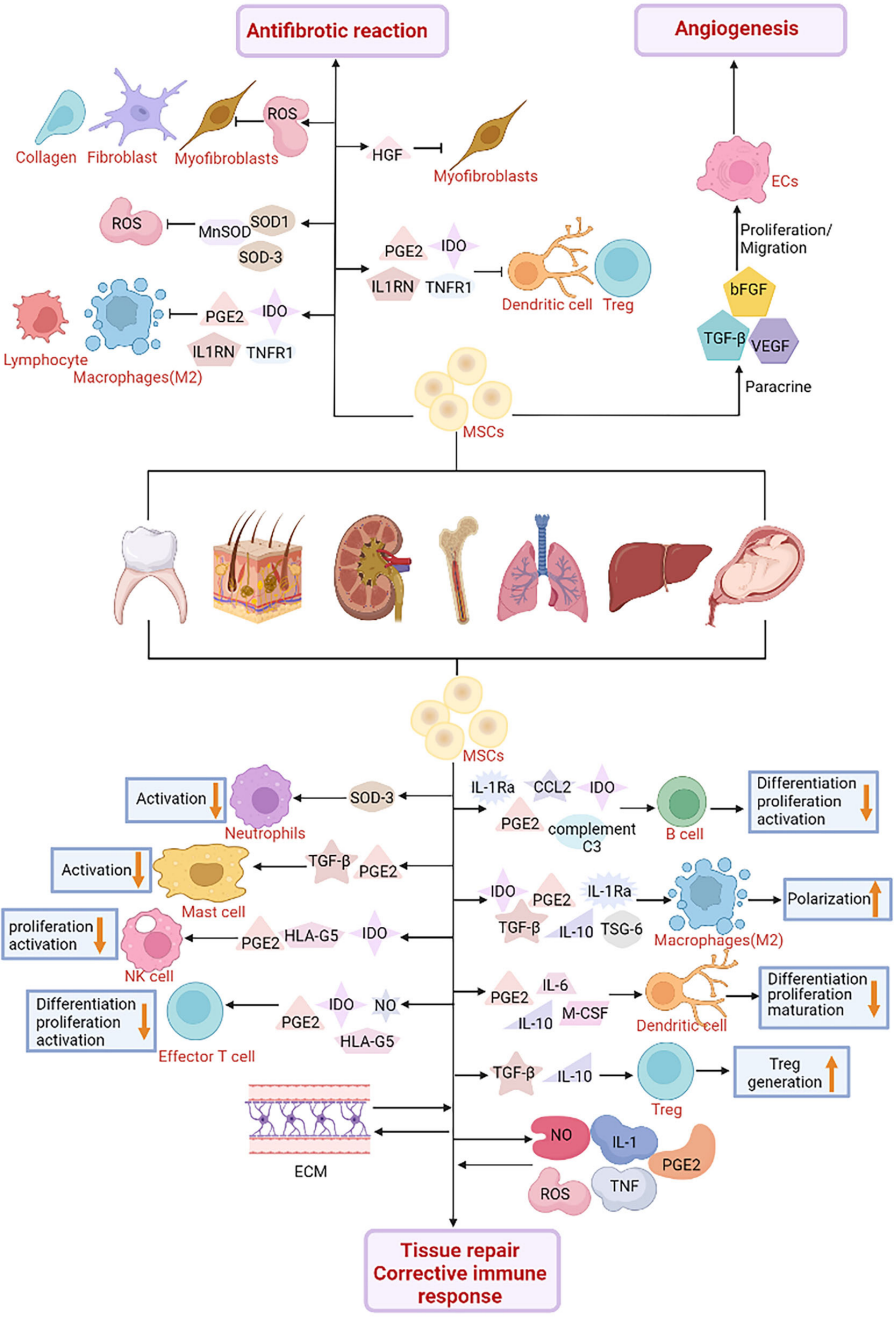
The role of Mesenchymal Stroma Cells (MSCs) in cell engineering technology. MSCs are used in cell therapy and might play a role in the treatment of diabetes. MSCs interact with immune cells such as natural killer cells (NK), macrophages, neutrophils, mast cells and dendritic cells. MSCs secrete a variety of soluble factors, Indoleamine 2, 3-dioxygenase (IDO), prostaglandin E2 (PGE2), C-C motif chemokine Ligand 2 (CCL2), IL-1 receptor antagonist (IL-1RA), complement C3, Tumor necrosis factor- (TNF) stimulated gene-6 (TSG-6), Transforming growth factor β (TGF-β), interleukin-10 (IL-10), interleukin-6 (IL-6), macrophage Colony stimulating factor (M-CSF) and human leukocyte antigen-G5 (HLA-G5) can inhibit the differentiation, proliferation and activation of various immune cell subgroups. Furthermore, MSCs interact with inflammatory factors including nitric oxide (NO), PGE2, tumor necrosis factor (TNF), reactive oxygen species (ROS), interleukin-1 (IL-1) and extracellular matrix (ECM) to promote tissue regeneration, repair and correct abnormal immune responses. In terms of the regulation of fibrosis microenvironment, MSCs release IDO, PGE2, interleukin-1 receptor antagonist (IL1RN), tumor necrosis factor receptor-1 (TNFR1) and other immunosuppressive factors to inhibit the activation of immune cells and the infiltration of inflammatory sites, and promote the formation of regulatory T (Treg) cells. In addition, MSCs produce antioxidant enzymes such as superoxide dismutase 1 (SOD1) and 3 (SOD3) and manganese superoxide dismutase (MnSOD) to reduce oxidative stress and reactive oxygen species levels. In addition, MSCs also secrete hepatocyte growth factor (HGF) and PGE2 in different ways to promote myofibroblast apoptosis and inhibit fibroblast proliferation and collagen production, respectively, thereby inhibiting the fibrotic response. It has also been found in diabetes studies that MSCs can secrete vascular endothelial growth factor (VEGF), basic fibroblast growth factor (bFGF), TGF-β and other angiogenic factors. Promotes the formation of new blood vessels and protects the islets from blood-mediated inflammatory response (IBMIR).

Transplantation of microencapsulated islets has been extensively studied as a promising treatment for type 1 diabetes. Meanwhile, challenges remain especially in achieving long-term function and reducing inflammation at the graft site that would lead to early islet dysfunction. Further, insufficient angiogenesis around graft sites remains a major issue resulting in malnutrition and hypoxia of encapsulated islets (113, 114). On the other hand, although iPSCs and ESCs have received sustained attention over the years, their clinical transformation is still hampered by ethical issues and risk of teratoma formation (115, 116). Thus, other feasible stem cells demand further investigation to overcome the obstacles mentioned above. Bone marrow mesenchymal stem cells are multipotent stem cells, which are mainly used for cell and regenerative therapy (117) (**Figure 4**). MSCs can secrete various immunomodulatory molecules, such as leukemia suppressor factor (LIF) (118), prostaglandin E2 (PGE2) (119), tumor necrosis factor (TNF)-stimulated gene 6 protein (TSG6) (120), and inhibit the infiltration of macrophages, neutrophils and monocytes into inflammatory sites by the release of TSG6. Besides, MSCs can restrict the fibrotic response by reducing myofibroblast differentiation and ECM deposition in fibroblasts and epithelial cells (121). In addition, MSCs also secrete angiogenic factors such as vascular endothelial growth factor (VEGF), basic fibroblast growth factor (bFGF) and transforming growth factor-β (TGF-β) (122). These secreted factors support islets to build their own vascular system. Thus, these characteristics confer MSCs the potential for supporting various functions when co-transplanted with islet cells. Earlier studies have demonstrated that MSCs and islet co-transplantation protect islets from problems associated with instant blood-mediated inflammatory response (IBMIR) and fatigue, diarrhea, and immune pneumonia caused by long-term use of immunosuppressive therapy (123–127). In the MSCs and islet co-transplantation, MSCs produce a microenvironment conducive to islet repair and longevity *in vitro* (128), and promote insulin secretion (129) and islet transplantation results in STZ-induced diabetic mice (130). Therefore, MSCs seem to be ideal supporting cells for co-transplantation with islets, although there are still research gaps to be filled soon.

As a common scaffold in tissue engineering, 3D cell culture system and hydrogel compositions can be applied to mammalian cells, such as islets. The small pore size of hydrogel prevents immune cells from passing through, thus protecting the islet from immune rejection, promoting the exchange of oxygen and nutrients, and improving the results of islet transplantation (131, 132). Some scholars embedded adipose tissue-derived mesenchymal stem cells (AT-MSCs) and islets into maleimide-dextrolic anhydride polymer hydrogels to evaluate the therapeutic effect of AT-MSCs in hydrogel composites on type 1 diabetic mice (133). *in vitro* experiments revealed that AT-MSCs significantly increased insulin secretion. After transplantation, blood glucose dropped from more than 400mg/dl to less than 150mg/dl within 4 days and remained stable until day 32, indicating prime results in treating type 1 diabetes.

A 3D structured cell transplantation platform called *CellSaic* was reported recently and it consisted of cells and petaloid pieces of a medical recombinant peptide (RCP) (134). Unlike traditional animal collagen, these petaloid-shaped pieces increased the surface area for cell adhesion and maintained empty spaces within the scaffold, which permitted substance-diffusion within the scaffold-cell aggregates. The vascular-inducing effect of MSC-*CellSaics* occured through altered release of various cytokines and growth factors, such as interleukin-8 (IL-8), bFGF and VEGF (135). Therefore, Kogawa’s team compared graft survival among three transplant conditions, islets, microencapsulated islets and microencapsulated combined with MSC-*CellSaics*. Transplant material remained in a mesh bag under the skin of diabetic mice until recovery (135). During 4 weeks following transplantation, blood glucose levels were significantly reduced and no inflammatory response was observed around the mesh bag within 14 days after transplantation in the MSC-*CellSaic* combined transplantation group compared with other groups. Thus, MSC-*CellSaics* could inhibit inflammation and immune rejection in early transplantation. Co-transplantation of MSC-*CellSaics* with encapsulated islets might be a more efficient approach to increase vascularization of grafts and mitigate inflammatory rejection of microencapsulated islets.

There is increasing evidence that layered slices of cells to construct 3D functional tissue through tissue engineering techniques can help maintain cellular function with nutrition (136). Herein, Hirabaru’s team used MSC-sheets as a support for subcutaneous islet transplantation into STZ-induced diabetic SCID mice. Only mice transplanted with islets cultured on MSC-sheets showed normalized blood glucose levels for at least 84 days after transplantation and increased neovascularization compared to islets grafted alone (137). Therefore, this new technical approach using MSC-sheets demonstrated a protective effect on islet survival and function.

Reconstructing a favorable microenvironment allowing integrin interactions could improve the survival rate of isolated islets (129). For instance, the addition of tripeptide arginine glycine aspartic acid (ARG-GLy-ASP, RGD) to microcapsules improves viability and function of C2 and C12 myoblasts (138). Based on these evidences, Laporte et al. developed a biocompatible composite capsule by combining M-rich alginate, RGD G-rich alginate and MSCs (139). This capsule composition improved the deleterious effect of encapsulation on human islets *in vitro*, showing decreased caspase activity and increased VEGF secretion. Improved islet outcome was possibly related to the cytoprotective function of MSCs whose paracrine effect was enhanced by the presence of RGD motif (140, 141). Therefore, MSCs and RGD G-rich alginate capsules could substantially ameliorate survival and function of encapsulated human islets *in vitro* although further studies are still needed to validate these results *in vivo*.

To date, it remains obscure whether pericapsular fibrosis overgrowth (PFO) occurring in an allograft environment could be alleviated after co-inclusion of MSCs and islet. PFO mainly involves macrophages and fibroblasts, which is related to the low survival rate of encapsulated islets (142). Therefore, islet co-encapsulation with MSCs was investigated as a strategy against PFO. It was previously validated that tumor necrosis factor-α (TNF-α) or interferon-γ (IFN-γ) can induce immunosuppressive activity of MSCs by producing cyclooxygenase-2 (Cox-2) and PGE2 before transplantation (143). Thus, Vaithilingam et al. used the co-encapsulation of islets with MSCs either unstimulated or stimulated by a mixture of cytokines IFN-γ and TNF-α to test PFO and islet survival. C57BL/6 mice were used for the transplantation with strong immune response and fibrotic response that were similar to that in humans. The results indicated that a slight decrease of PFO in the stimulated mice was sufficient to significantly improve graft survival and islet activity (144). Immunosuppression in the stimulated MSC group was correlated to increased production of NO, which played a major role in regulating T cell immune response (145, 146). However, prior to clinical translation for patients with T1DM, further studies should be conducted using stimulated human bone-derived MSCs (BMSCs) co-encapsulated together with human islets in allograft settings using humanized mouse models.

## Pancreatic Progenitor Cell as Another Resource of β-cells

Progenitor cells are considered as cells which have the ability to differentiate into a specific target cell. Studies have shown that β-cell progenitor cells derived from human embryonic stem cells express high levels of NKX6-1 and are prone to further mature into glucose-responsive β-cells (76). The key difference between progenitor cells and stem cells is that stem cells have an unlimited proliferation capacity, whereas progenitor cells can divide only a limited number of times (147). There are several theories about the origin of pancreatic progenitor cells. Mostly accepted is that islet progenitor cells are derived from pancreatic ducts, where they regenerate, differentiate and migrate to form new islets (148, 149). Studies have shown that islet formation starts early in embryonic development after birth, through the migration of pancreatic primordial cells out of epithelial ducts to form clusters of epithelial cells. Later-on these cells differentiate then into hormone-producing endocrine cells (150, 151). Formation of new islets, meaning the differentiation of islet progenitor cells into new islets, in or near ducts, has long been considered as an active process occurring after birth (152, 153). In a genealogy-tracing experiment to genetically label duct cells, the Cre-Lox system, in which Cre recombinase expression was driven by the promoter of carbonic anhydrase II (CAII), a marker of mature ducts cells, was used. Thus, pancreatic duct cells expressing CAII, have been shown to generate new islets as well as acini after birth and injury (154, 155). In addition, another potential source for β-cells is the islet itself. *Ex vivo* proliferation of β-cells or the plasticity of α-cells are still interesting concepts for generating β-cells (156). In conclusion, islet progenitor cells may exist not only in ductal epithelium, but also in islets itself (148, 157).

Several *in vitro* studies have shown that insulin-producing cells can be differentiated from adult pancreatic duct tissues (158, 159). Bonner-Weir et al. cultured adult duct tissue with matrix gel and observed islet buds composed of cytokeratin 19 (CK19) positive duct cells and insulin-positive cells (72). Other studies have also shown that some CK19-positive ductal epithelial cells differentiate into endocrine cells (160). Also, progenitor-like cells isolated from the adult pancreas formed tubular mature annular/dense colonies expressing PDX-1 and SOX9 and differentiated into endocrine/acinar colonies. Most endocrine/acinar colonies contained a majority of β-like cells which expressed and secreted insulin and C-peptide (161, 162). Further, duct cells under the action of glucagon-like peptide-1 (GLP-1) differentiate into islet endocrine cells including β-cells *in vitro* (163, 164). In addition, it has been early substantiated that β-cells are regenerated by duct cell trans-differentiation. Islet and pancreatic regeneration are achieved by replication of β-cells near or inside the pancreatic ducts, or by progenitor cells expressing Ngn-3 (165, 166).

Interestingly, progenitor cells from outside of the pancreas, such as murine skeletal muscle-derived progenitor cells, have been differentiated into insulin-producing clusters by a differentiation protocol comprising four steps of culture. These progenitor cells transformed into mature β-cells during development and significantly reduced hyperglycemia and improved survival after transplantation into STZ-induced diabetic mice (167). Moreover, Yi Arial Zeng’s team identified a new population of protein C receptor-positive (Procr+) endocrine progenitor cells by single-cell RNA sequencing and that did not express known endocrine or exocrine differentiation markers of the adult mouse pancreas (168). In clonal density culture, islets can be formed stably, thus exerting its hypoglycemic function *in vivo*. It was also found that transplantation of pancreatic progenitor cells under the mammary fat pad or renal capsule did not affect their eventual differentiation into functional β-cells, despite no exposure to the “pancreatic” microenvironment (169). In conclusion, pancreatic progenitor cells capable of forming islet-like structures *in vivo* derived from human pluripotent stem cells represent a potential cell source for the treatment of type I diabetes.

## Discussion and Summary

Worldwide, it is estimated that there are currently 463 million persons with diabetes and this number is projected to reach 578 million by 2030, and 700 million by 2045. About 10 percent of those have T1DM, while type 2 diabetes mellitus (T2DM) is the most common type of diabetes that accounts for about 90 percent of all diabetes cases. With a variety of common and predisposing complications, 10% of global health expenditure (USD 760 billion) is spent on the prevention and cure of diabetes.

In this review, we expose recent advances in the development and use of pancreatic progenitor cells, bone marrow stem cells (MSCs), embryonic stem cells (ESCs) and pluripotent stem cells (PSCs) as cell sources for engineering islets for future β-cell replacement therapies, with a focus on recent biotechnology engineering. Although a significant progress has been achieved for the development of islet clusters from human stem cells, only a few practical applications of bioengineering technology are realized in the treatment of diabetes. For the treatment of T1DM, novel attempts have focused on the development of bioengineering strategies such as microencapsulation with the aim to avoid immunosuppressive agents (170). For the treatment of T2DM, the exploration for new treatments includes stem cell differentiation, drug therapy and other methods (171). Although autologous iPSCs could theoretically be optimal for clinical use by avoiding immune rejection, long-term results of iPSC-differentiated cells transplantation still needs further confirmation.

Besides considerable advances made in the field, such as improved protocols for endocrine differentiation from SC, validation of therapeutic efficiency within animals and humans remains limited. Another challenge in this field is the lack of allogeneic and autoimmune humanized T1DM models to study the efficacy and safety of various stem cell therapy or devices. Given the major challenge remaining for the clinical applications, the hurdles include immune rejection, recurrence of autoimmunity, genetic stability and risk of tumorigenesis. Genetic instability could aggravate the risk of tumorigenicity, while *de novo* mutations in mtDNA obtained from reprogramming to the iPSCs stage, long-term culture and differentiation into target cells and the immune environment of the transplantation site could activate immune response in autologous transplantation. However, evidence on the efficacy of immunosuppressive molecules in suppressing allogeneic immune responses remains obscure. Programmed death ligand-1 (PD-L1), a member of the CD28 T cell family, binds to programmed cell death (PD-1), and this could downregulate T cell proliferation and inhibit immune responses, which hypothetically prevents allograft rejection in organ transplantation (172). PD-L1 knockout caused the acceleration of cardiac allograft rejection in animal models, while clinical data from endomyocardial transplant biopsies and explant hearts indicated that the relative reduction of PD-L1 expression compared with PD-1 could be a defining pathologic feature (173). Cytotoxic T lymphocyte antigen 4-immunoglobulin fusion protein (CTLA4-Ig) blocks T cell co-stimulatory pathways while PD-L1 activates T cell inhibitory pathway, thus they function jointly in maintaining peripheral tolerance by suppressing T cell activity. The joint knock-in of CTLA4-Ig and PD-L1 in human embryonic stem cells (hESCs) successfully led to the immunoprotection of hESCs-derived teratomas, fibroblasts, and cardiomyocytes in humanized mice (Hu-mice) (174). Besides, PD‐1/PD‐L1 checkpoint axis is substantiated with its predominant role in regulating immune response in human heart transplant recipients and a mouse model of heart transplant rejection. Reduced graft endothelial PD-L1 expression was negatively relevant to the proportion of CD8 +T-cell infiltration in human heart transplantation, meanwhile, the abrogation of graft endothelial PD-L1 expression may facilitate acute rejection and lead to decreased graft survival (175). Therefore, the protective strategies addressing immune response without requiring systemic immune suppression are urgent to be developed. Herein, systemic use of exogenous PD‐L1‐Ig, overexpression of PD‐L1 in transplanted cells and tissue overexpression of PD‐L1 before transplantation are three promising strategies in preclinical induction of immune tolerance with PD‐1 signaling (176). Furthermore, clinical data indicated the considerable relevance between PD-L1 expression in HSCs and degree of T cell apoptosis, which conferred further research potential in allogeneic transplantation of HSCs (177).

Future countermeasures and therapy strategies may benefit from a better knowledge of molecular pathways that affect immune conditions, such as the PD1/PD-L1 checkpoint axis, contribute to reducing graft rejection risk of patients, and effectively promote the development of stem cell-derived therapies. Also, ongoing research is needed to stimulate bioengineering technologies toward long-term functional medical devises for the radical cure of diabetes, and benefiting diabetic patients.

## Author Contributions

LB and YW conceived and designed the review. SD and YL wrote the manuscript. QZ prepared the figures. LB, CG-G and YW reviewed and edited the manuscript. All authors read and approved the final manuscript.

## Funding

This study was sponsored by the National Science Foundation of China (81802504), the grant from Sichuan Medical Association (Q19037) and the grant from Chengdu Science and Technology Bureau (2021-YF05-00225-SN) for YW. CG-G and LB are supported by the Insuleman foundation, Child foundation and the foundation la Colombe. YW and LB are supported by Sichuan Science and Technology Bureau (No. 2022YFT0005 and 2019YFS0439). This study is also supported by grants from Health Commission of Sichuan Province (No.[2019]30), from Sichuan Administration of Traditional Chinese Medicine (No. 2020JC0114) and from Beijing Xisike Clinical Oncology Research (No. Y-QL202101-0125).

## Conflict of Interest

The authors declare that the research was conducted in the absence of any commercial or financial relationships that could be construed as a potential conflict of interest.

## Publisher’s Note

All claims expressed in this article are solely those of the authors and do not necessarily represent those of their affiliated organizations, or those of the publisher, the editors and the reviewers. Any product that may be evaluated in this article, or claim that may be made by its manufacturer, is not guaranteed or endorsed by the publisher.
